# COPD burden trends in China and G20 countries from GBD 2023 and Mendelian randomization

**DOI:** 10.1016/j.isci.2026.117058

**Published:** 2026-08-10

**Authors:** Hu Shiyu, Tan Xiaoli, Zhao Miaomiao, Chen Wenyu

**Affiliations:** 1 Department of Respiratory Medicine, Affiliated Hospital of Jiaxing University, Jiaxing 314000, China; 2 Zhejiang Chinese Medical University, Hangzhou 310000, China

**Keywords:** chronic obstructive pulmonary disease, Global Burden of Disease, joinpoint regression, forecasting, Mendelian randomization

## Abstract

Chronic obstructive pulmonary disease (COPD) remains a major contributor to morbidity and mortality, making comparative evidence on long-term burden and risk factors important for prevention. Using the Global Burden of Disease (GBD) 2023 data, we assessed COPD prevalence, incidence, deaths, disability-adjusted life years (DALYs), years lived with disability (YLDs), years of life lost (YLLs), and age-standardized rates (ASRs) in China and the Group of Twenty (G20) countries from 1990–2023. Joinpoint regression characterized temporal changes, autoregressive integrated moving average (ARIMA) models projected burden to 2028, and two-sample Mendelian randomization evaluated genetically proxied risk factors in human populations. In 2023, China had substantial COPD burden, with 52.5 million prevalent cases and nearly 1.0 million deaths. Absolute burden increased since 1990, whereas ASRs generally declined across China and G20 countries. Fifteen exposures showed Bonferroni-corrected associations with COPD risk. These findings highlight persistent disparities in COPD burden and support targeted prevention while recognizing that genetic evidence requires further validation.

## Highlights

• GBD 2023 data compared 1990–2023 COPD burden in China and G20 countries
• ARIMA models projected COPD burden trends to 2035
• Cases and deaths rose, yet age-standardized rates fell; China exceeded G20 average
• MR identified 15 causal factors to inform COPD prevention strategies

## Graphical abstract

**Figure:**
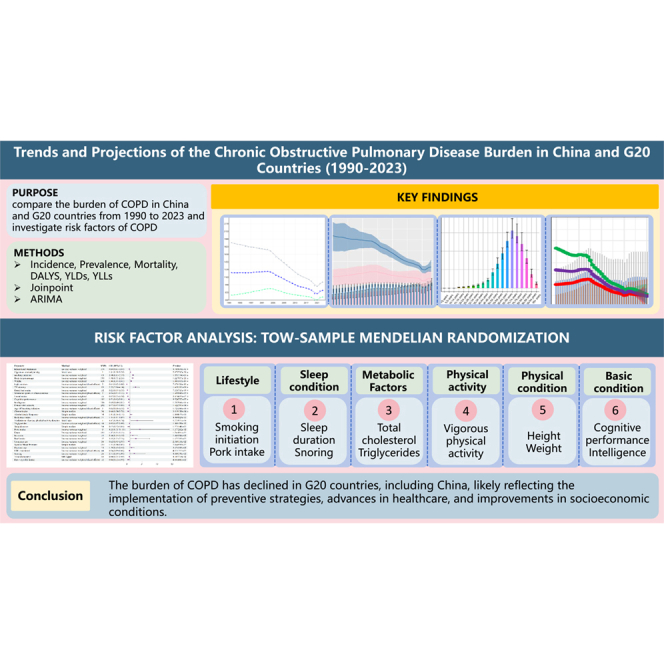

## Teaser

Health sciences; Medicine; Medical specialty; Internal medicine; Respiratory medicine; Public health

## Introduction

Chronic obstructive pulmonary disease (COPD) is a highly burdensome chronic respiratory disorder. In 2023, there were approximately 214.6 million prevalent cases of COPD worldwide, accounting for 82.3% of the deaths due to all chronic respiratory diseases (CRDs).^1^ Over the next two decades, the burden of COPD is projected to continue rising.^2^ China is among the countries bearing a particularly heavy COPD burden. In 2021, China reported approximately 50.6 million prevalent cases, 4.4 million incident cases, 1.29 million deaths, and 23.6 million disability-adjusted life years (DALYs) attributable to COPD.^3^ Examining disparities between China and other countries is therefore of substantial importance for informing public health policies and optimizing healthcare strategies.^4^

Heterogeneity in COPD, drivers ranging from tobacco use, population aging, and long-term exposure to air pollution in high-income countries to household air pollution, occupational exposures, delayed diagnosis, and uneven healthcare access in emerging economies, provides an opportunity to better understand how different demographic, environmental, and health-system contexts shape COPD burden through cross-national comparison.^5^ The Group of Twenty (G20), comprising both high-income and emerging economies, accounts for approximately 85% of global gross domestic product, over 75% of global trade, and about two-thirds of the world’s population.^4^ Although the G20 is not a homogeneous epidemiological or climatic region, its members represent major economies with substantial differences in population structure, exposure profiles, healthcare capacity, and data quality. Therefore, comparison within the G20 framework can provide a broad and heterogeneous reference for contextualizing China’s COPD burden. China’s dual status as a G20 member and the largest developing country presents unique challenges for COPD prevention and management. Rapid population aging, historically high smoking prevalence among men, ambient air pollution, occupational exposures, and regional disparities in healthcare access have jointly contributed to the persistent COPD burden in China.^3^ At the same time, China has experienced rapid socioeconomic development and health-system expansion, making its COPD trajectory distinct from both high-income countries with more mature chronic disease management systems and emerging economies facing similar environmental and healthcare-access challenges. Existing comparative studies have often focused on global trends, individual countries, or broad income-based groups, which may overlook China’s distinctive position among major economies.^6^^,^^7^ A comparative analysis of COPD burden across China and G20 countries may therefore help clarify China’s relative burden, identify differences in temporal trends, and provide evidence for more context-specific prevention and control strategies.

In 2025, the Global Burden of Disease (GBD) study underwent a major update, extending data coverage through 2023 and documenting changes in global disease burden before and after the COVID-19 pandemic, the COVID-19 pandemic may have affected COPD management through multiple pathways, including disruption of routine outpatient care, reduced access to pulmonary rehabilitation, delayed spirometry-based diagnosis, interruption of regular medication follow-up, and postponed treatment for acute exacerbations. In addition, patients with COPD may have avoided or delayed medical visits because of concerns about viral exposure, while healthcare systems faced substantial pressure during pandemic waves. Compared with previous GBD rounds, GBD 2023 re-estimated the full time series of disease burden using newly available data sources and updated modeling frameworks. GBD 2023 also updated the comparative risk assessment framework for risk-factor attribution, evaluating the burden attributable to 88 modifiable risk factors across 159 health outcomes. For COPD, this enabled updated estimation of the burden attributable to major established risk factors, such as smoking, ambient particulate matter pollution, household air pollution, occupational exposures, and other relevant environmental risks. In addition, GBD 2023 included methodological refinements in cause-of-death estimation during the COVID-19 era, including corrections for potential misclassification of COVID-19-related deaths, updates to COVID-19 mortality estimation, and updates to the Cause of Death Ensemble model framework. These changes are important for interpreting recent fluctuations in COPD mortality and DALY estimates after 2020.^8^^,^^9^ Based on GBD 2023, the present study comprehensively compares and analyzes the burden of COPD in China and G20 countries from 1990–2023. In addition, we conducted a two-sample Mendelian randomization (MR) analysis to investigate the causal relationships between multiple potential risk factors and COPD. By systematically examining temporal trends and causal determinants, this study aims to provide up-to-date evidence to support policymakers in developing more effective prevention and control strategies.

## Results

### Overall trends of COPD prevalence, incidence, mortality, YLDs, YLLs, and DALYs in China and G20 countries

Overall, the number of prevalent COPD cases in China increased from 24,220,063.00 (95% uncertainty interval [UI]: 21,833,790–26,557,597) in 1990 to 52,490,910.07 (95% UI: 45,703,702.19–58,446,498.40) in 2023, representing a 116.72% increase. Incident cases rose from 2,238,997.72 (95% UI: 2,055,481.25–2,405,044.47) to 4,440,422.11 (95% UI: 3,956,324.12–4,921,625.41). During the same period, the cumulative number of COPD-related deaths increased by 46.86%, reaching 998,236.70 (95% UI: 770,081.36–1,436,257.71) in 2023. DALYs increased from 14,534,512.32 (95% UI: 9,732,302.70–18,769,915.19) in 1990 to 17,281,132.76 (95% UI: 14,253,963.93–23,791,494.91) in 2023, corresponding to an 18.90% rise. In 2023, the numbers of years lived with disability (YLDs) and years of life lost (YLLs) were 2,326,926.11 (95% UI: 1,857,158.28–2,861,621.75) and 14,954,206.65 (95% UI: 12,014,749.06–21,442,080.24), respectively. Despite the marked increase in absolute case numbers, age-standardized rates (ASRs) declined over the study period. From 1990–2023, the estimated annual percent change (EAPC) was −0.65 (95% confidence interval [CI]: −0.75 to −0.55) for the ASPR, −1.16 (95% CI: −1.25 to −1.07) for the age-standardized incidence rate (ASIR), −3.26 (95% CI: −3.74 to −2.78) for the age-standardized mortality rate (ASMR), and −3.45 (95% CI: −3.89 to −3.00) for the age-standardized DALY rate (ASDR). The EAPCs for age-standardized YLD and YLL rates were −0.86 (95% CI: −0.98 to −0.74) and −3.72 (95% CI: −4.20 to −3.23), respectively. In comparison, in 1990, the G20 countries exhibited lower ASRs—including age-standardized prevalence rate (ASPR), ASIR, ASMR, ASDR, and age-standardized YLL rates—than China. However, by 2023, the ASPR, ASDR, and age-standardized YLL rates in G20 countries had exceeded those observed in China (Table 1).

**Table 1:** All-age cases, age-standardized incidence, mortality, YLDs, YLLs, DALYs, and corresponding EAPCs of COPD in China and G20 Countries in 1990 and 2023

Location	Measure	1990		2023		
		All-age cases	ASR	All-age cases	ASR	1990–2023 EAPC
		95% UI	95% UI	95% UI	95% UI	95% UI
China	incidence	2238997.72 (2055481.25–2405044.47)	275.52 (254.92–292.37)	4440422.11 (3956324.12–4921625.41)	197.76 (178.00–216.92)	−1.16 (−1.25–−1.07)
	prevalence	24220063.00 (21833790.01–26557596.97)	2870.24 (2578.13–3151.49)	52490910.07 (45703702.19–58446498.40)	2367.20 (2077.16–2616.02)	−0.65 (−0.75–−0.55)
	mortality	679717.58 (443316.62–884105.84)	112.79 (75.84–147.05)	998236.70 (770081.36–1436257.71)	46.60 (35.86–67.03)	−3.26 (−3.74–−2.78)
	YLDs	1125835.58 (926623.70–1359018.62)	131.77 (108.71–158.85)	2326926.11 (1857158.28–2861621.75)	105.14 (84.49–127.67)	−0.86 (−0.98–−0.74)
	YLLs	13408676.74 (8621130.68–17588219.40)	1868.85 (1225.59–2444.85)	14954206.65 (12014749.06–21442080.24)	672.71 (537.94–955.92)	−3.72 (−4.2–−3.23)
	DALYs	14534512.32 (9732302.70–18769915.19)	2000.61 (1357.70–2580.27)	17281132.76 (14253963.93–23791494.91)	777.86 (642.58–1057.98)	−3.45 (−3.89–−3)
G20	incidence	5990884.69 (5438612.50–6431444.60)	216.34 (196.84–232.30)	12529984.17 (11400238.84–13608513.29)	197.68 (180.81–213.46)	−0.32 (−0.38–−0.26)
	prevalence	73230291.77 (65820137.33–79852277.67)	2681.31 (2411.07–2928.74)	159108704.90 (143049075.20–172766870.90)	2516.10 (2269.60–2727.42)	−0.19 (−0.26–−0.12)
	mortality	1433700.65 (1068364.41–1699125.21)	58.57 (44.09–68.92)	2673864.35 (2290346.44–3259973.00)	41.65 (35.61–50.77)	−1.41 (−1.68–−1.14)
	YLDs	4773997.99 (4027920.67–5492948.21)	172.12 (145.56–199.20)	10617894.56 (8913630.84–12417300.92)	169.21 (141.93–197.45)	−0.09 (−0.19– 0.02)
	YLLs	28920719.99 (21546887.88–34321500.79)	1092.69 (814.90–1294.32)	45855297.09 (39983112.99–55435958.26)	710.05 (618.31–855.69)	−1.71 (−1.97–−1.46)
	DALYs	33694717.98 (26280434.37–38848902.30)	1264.81 (992.11–1455.58)	56473191.65 (49706511.18–65973822.01)	879.26 (774.70–1025.12)	−1.44 (−1.67–−1.22)

COPD, chronic obstructive pulmonary disease; G20, Group of 20; DALYs, disability-adjusted life years; YLDs, years lived with disability; YLLs, years of life lost; ASR, age-standardized rate.

### Joinpoint regression analysis of COPD burden in China and G20 countries

**Figure 1:**
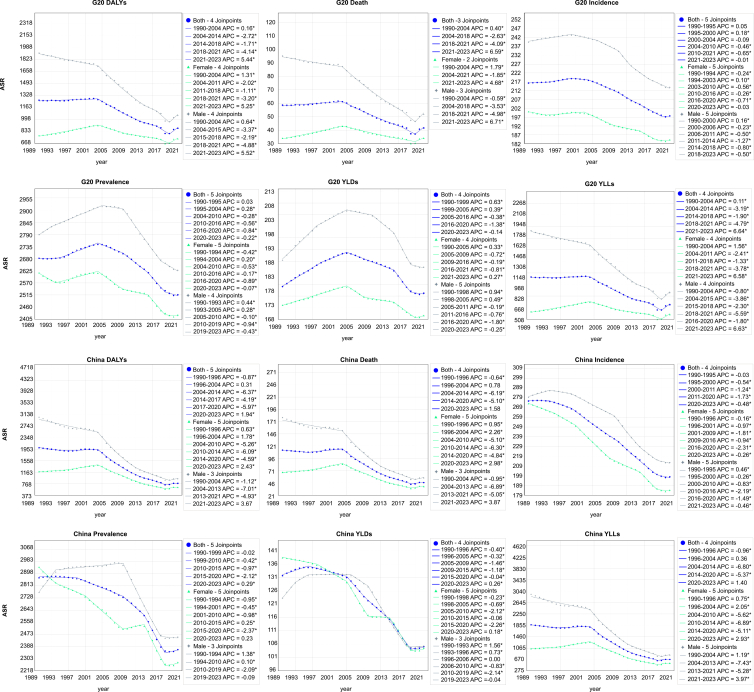
Joinpoint regression analysis of DALYs, ASMR, ASIR, ASPR, and age-standardized YLD and YLL rates of COPD in China and G20 countries from 1990 to 2023 Asterisks indicate a *p* value less than 0.05. Gray lines indicate estimates for both sexes, blue lines indicate estimates for males, and green lines indicate estimates for females. COPD, chronic obstructive pulmonary disease; G20, Group of 20; DALYs, disability-adjusted life years; YLDs, years lived with disability; YLLs, years of life lost; ASRs, age-standardized rates.

Joinpoint regression analyses of the ASIR, ASMR, ASDR, and age-standardized YLD and YLL rates for COPD in China and G20 countries from 1990–2023 are presented in Figure 1. In China, ASPR showed an overall downward trend from 1990–2020, with the decline becoming progressively more pronounced over time. Specifically, the annual percent change (APC) in ASPR was −0.02 (*p* > 0.05) during 1990–1999, −0.42 during 1999–2010, −0.97 during 2010–2015, and −2.12 during 2015–2020 (*p* < 0.05). However, from 2020–2023, a slight rebound was observed (APC = 0.29, *p* < 0.05). For ASIR, a consistent downward trend was observed from 1995–2023 (1995–2000: APC = −0.54; 2000–2011: APC = −1.24; 2011–2020: APC = −1.73; 2020–2023: APC = −0.48; all *p* < 0.05). ASMR increased between 1996 and 2004 (APC = 0.78, *p* < 0.05), followed by a sustained decline from 2004–2020 (2004–2014: APC = −6.19; 2014–2020: APC = −5.10; *p* < 0.05). ASDR declined during 1990–1996 and 2004–2020 (1990–1996: APC = −0.87; 2004–2014: APC = −6.37; 2014–2017: APC = −4.19; 2017–2020: APC = −5.97; *p* < 0.05), but increased from 2020–2023 (APC = 1.94, *p* < 0.05). Age-standardized YLD rates decreased from 1996–2020 (1996–2005: APC = −0.32; 2005–2009: APC = −1.46; 2009–2015: APC = −1.18; 2015–2020: APC = −2.04), whereas increasing trends were observed during 1990–1996 (APC = 0.40) and 2020–2023 (APC = 0.26). Age-standardized YLL rates declined during 1990–1996 and 2004–2020 (1990–1996: APC = −0.96; 2004–2014: APC = −6.80; 2014–2020: APC = −5.37), while remaining relatively stable during 1996–2004 and 2020–2023 (*p* > 0.05). In G20 countries, ASPR increased from 1995–2004 (APC = 0.28, *p* < 0.05), followed by a sustained decline from 2004–2023 (2004–2010: APC = −0.28; 2010–2016: APC = −0.56; 2016–2020: APC = −0.84; 2020–2023: APC = −0.22; *p* < 0.05). ASIR increased during 1995–2000 (APC = 0.18, *p* < 0.05) but declined from 2004–2021 (2004–2010: APC = −0.46; 2010–2021: APC = −0.65; *p* < 0.05). Both ASMR and ASDR in G20 countries showed downward trends from 2004–2021 (ASMR: 2004–2018: APC = −2.63; 2018–2021: APC = −4.09; ASDR: 2004–2014: APC = −2.72; 2014–2018: APC = −1.71; 2018–2021: APC = −4.14; *p* < 0.05). In contrast, increasing trends were observed during 1990–2004 and 2021–2023 (ASMR: 19902004: APC = 0.40; 2021–2023: APC = 6.59; ASDR: 1990–2004: APC = 0.16; 2021–2023: APC = 5.44; *p* < 0.05). Age-standardized YLD rates in G20 countries increased from 1990–2005 (1990–1999: APC = 0.63; 1999–2005: APC = 0.39; *p* < 0.05), followed by a decline from 2005–2020 (2005–2016: APC = −0.38; 2016–2020: APC = −1.38; *p* < 0.05). Age-standardized YLL rates declined from 2004–2021 (2004–2014: APC = −3.19; 2014–2018: APC = −1.90; 2018–2021: APC = −4.79; *p* < 0.05), whereas increasing trends were observed during 1990–2004 (APC = 0.11) and 2021–2023 (APC = 6.64; *p* < 0.05).

### Trends in the burden of COPD in China and G20 countries

**Figure 2:**
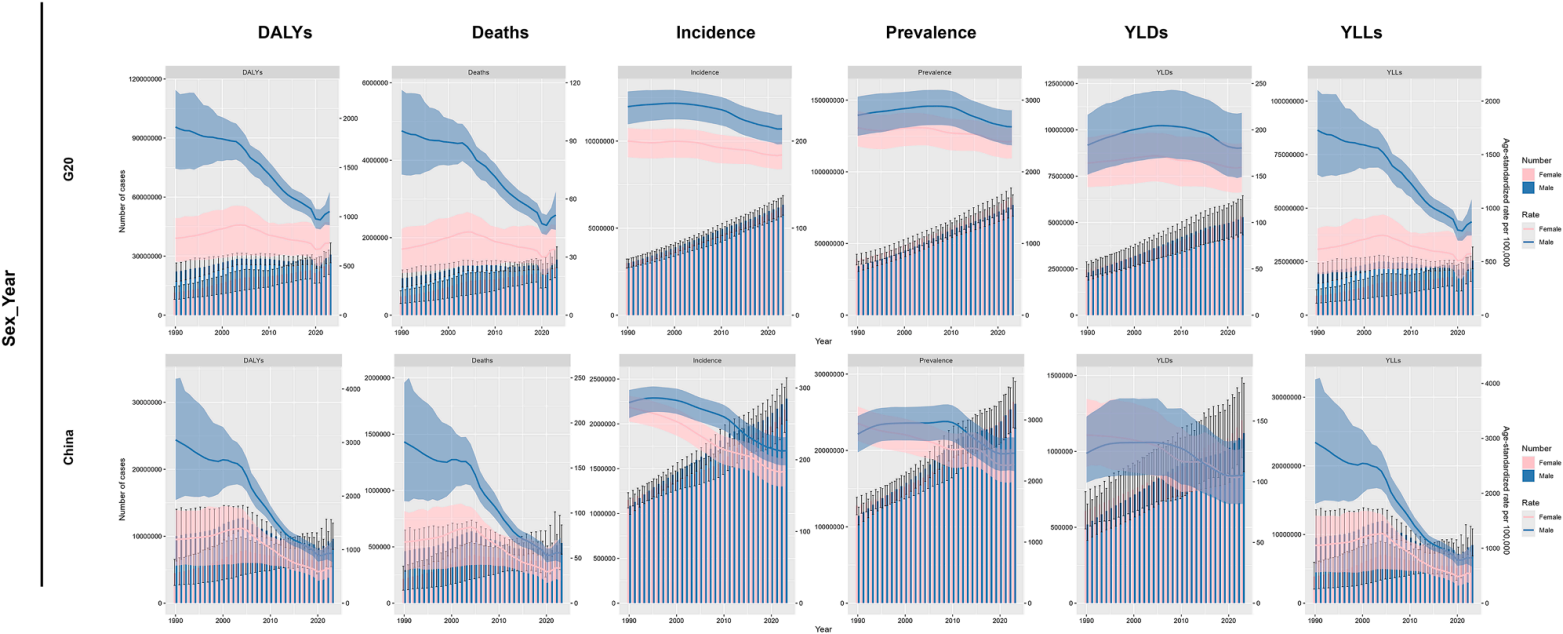
Comparison of the trends in DALYs, ASMR, ASIR, ASPR, and age-standardized YLD and YLL rates of COPD in China and G20 countries from 1990 to 2023 Bars represent the absolute number of cases and correspond to the left *y* axis. Lines represent age-standardized rates per 100,000 population and correspond to the right *y* axis. Error bars and shaded bands indicate uncertainty intervals. Color coding: blue indicates males and pink indicates females. COPD, chronic obstructive pulmonary disease; G20, Group of 20; DALYs, disability-adjusted life years; YLDs, years lived with disability; YLLs, years of life lost; ASRs, age-standardized rates.

Figure 2 illustrates temporal changes in the COPD burden in China and G20 countries from 1990–2023, including the absolute numbers and ASR of DALYs, deaths, incidence, prevalence, YLDs, and YLLs, stratified by sex. Overall, although some fluctuations were observed across calendar years, the trends were largely consistent. The absolute numbers of incident and prevalent cases increased steadily in both China and the G20 countries, despite a gradual decline—or an initial plateau followed by a decrease—in ASIR and ASPR. In contrast, the ASRs of deaths, YLLs, and DALYs demonstrated marked downward trends, with a more pronounced decline observed in China. However, the corresponding absolute numbers showed only modest reductions and exhibited a slight rebound in the most recent years. Regarding non-fatal burden, the absolute number of YLDs increased over time, whereas the corresponding ASR initially rose and then declined. In terms of sex differences, males consistently exhibited higher ASRs across all indicators compared with females, with the disparity being particularly pronounced for deaths, YLLs, and DALYs. This sex-specific pattern was consistent with the overall trends observed in the G20 countries.

### Sensitivity analysis

Based on the 1990–2019 pre-pandemic trend, the projected 2023 age-standardized incidence, prevalence, mortality, YLD, YLL, and DALY rates in China were 187.3402, 2143.8252, 37.3509, 99.6457, 545.3056, and 643.3512 per 100,000 population, respectively. However, the corresponding observed GBD 2023 estimates were higher, at 197.7558, 2367.1972, 46.6047, 105.1447, 672.7117, and 777.8564 per 100,000 population, respectively. Similarly, for the G20 countries, the projected 2023 age-standardized incidence, prevalence, mortality, YLD, YLL, and DALY rates based on the 1990–2019 trend were 195.2993, 2471.2266, 36.9487, 163.0909, 632.9227, and 795.4125 per 100,000 population, respectively, and the observed 2023 value were consistently higher than these projected values(Figures S1–S3).

### Disaggregate G20 analysis

Disaggregated analysis of selected G20 countries revealed substantial heterogeneity that was obscured by the overall G20 average. China showed a marked decline in DALY, death, and YLL rates from 1990–2021. Compared with emerging economies, China achieved a faster and larger reduction than India and Brazil; India consistently had the highest burden, while Brazil showed an intermediate pattern with declining mortality-related indicators but relatively stable incidence and prevalence. In contrast, high-income countries such as Germany and Japan generally maintained lower DALY, death, and YLL rates (Figure S4).

### Comparison of COPD burden among different age groups in China and G20 countries

**Figure 3:**
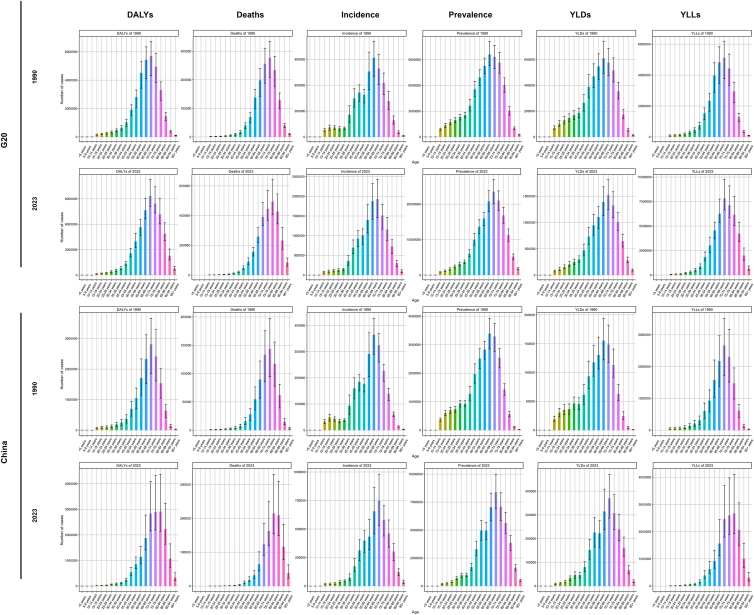
Number of DALYs, mortality, incidence, prevalence, YLD and YLL cases for different age groups of COPD in China and G20 countries in 1990 and 2023 Bars represent the number of cases in each age group, and vertical error bars indicate uncertainty intervals. The *x* axis shows age groups, and the *y* axis shows the corresponding number of cases. Bar colors represent different age groups. COPD, chronic obstructive pulmonary disease; G20, Group of 20; DALYs, disability-adjusted life years; YLDs, years lived with disability; YLLs, years of life lost; ASRs, age-standardized rates.

**Figure 4:**
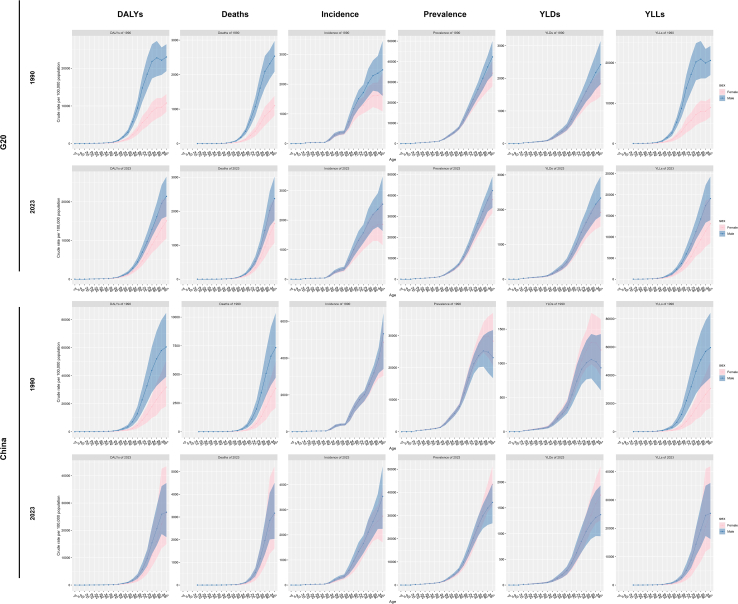
Comparison of DALYs, ASMR, ASIR, ASPR, and age-standardized YLD and YLL rates for different age groups of COPD in China and G20 countries in 1990 and 2023 Lines indicate age-specific rates per 100,000 population, and shaded bands represent the corresponding uncertainty intervals. Blue indicates males, and pink indicates females. COPD, chronic obstructive pulmonary disease; G20, Group of 20; DALYs, disability-adjusted life years; YLDs, years lived with disability; YLLs, years of life lost; ASRs, age-standardized rates.

Figures 3 and 4 present the numbers (Figure 3) and corresponding rates (Figure 4) of DALYs, deaths, incident cases, prevalent cases, YLDs, and YLLs attributable to COPD across different age groups in China and G20 countries in 1990 and 2023. In 2023, in China, the numbers of DALYs, deaths, and YLLs peaked in the 80–84-year age group, whereas the numbers of incident cases, prevalent cases, and YLDs reached their highest levels in the 70–74-year age group.

### Prediction of incidence, prevalence, mortality, YLD, YLL and DALY rates of COPD in China and G20 countries from 2024–2028

**Figure 5:**
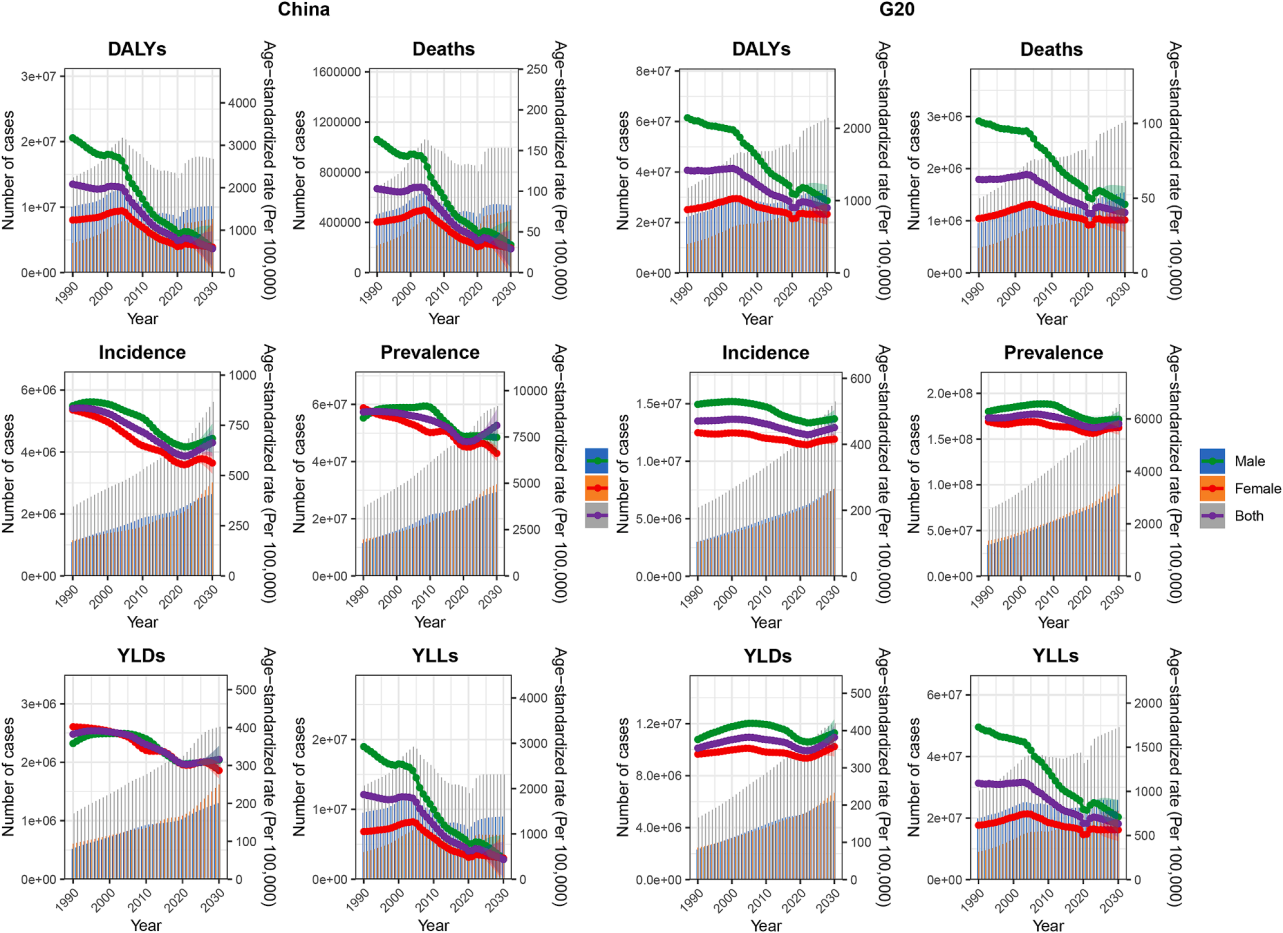
Temporal trends of DALYs, ASMR, ASIR, ASPR, and age-standardized YLD and YLL rates of COPD in China and G20 countries from 1990 to 2023, with projections for 2024–2028 Bars indicate the absolute number of cases and correspond to the left *y* axis, whereas lines indicate age-standardized rates per 100,000 population and correspond to the right *y* axis. Error bars represent uncertainty intervals for the number of cases. Green indicates males, red indicates females, and purple indicates both sexes. COPD, chronic obstructive pulmonary disease; G20, Group of 20; DALYs, disability-adjusted life years; YLDs, years lived with disability; YLLs, years of life lost; ASRs, age-standardized rates.

We applied autoregressive integrated moving average (ARIMA) models to fit sex-specific time series of burden for COPD from 1990–2023. Future trends from 2024–2028 were subsequently projected (Figure 5 and Table 2). In China, the projected incidence rate among males is expected to increase from 213.72 per 100,000 in 2024 to 221.73 per 100,000 in 2028, while among females it is projected to rise from 186.79 per 100,000 to 191.67 per 100,000. Overall, the incidence rate is anticipated to increase from 200.16 per 100,000 to 213.14 per 100,000. In G20 countries, the incidence rate among males is projected to increase from 214.69 per 100,000 to 217.81 per 100,000, and among females from 185.84 per 100,000 to 189.97 per 100,000. The overall incidence rate is projected to rise from 199.03 per 100,000 to 204.43 per 100,000. For mortality rate, in China, the mortality rate among males is projected to decline from 53.87 per 100,000 to 43.67 per 100,000 by 2028, while among females it is expected to decline from 37.71 per 100,000 to 34.61 per 100,000. The overall mortality rate is projected to decline from 43.97 per 100,000 to 35.84 per 100,000. In G20 countries, male mortality rate is projected to decrease from 50.75 per 100,000 to 45.56 per 100,000, whereas female mortality rate is expected to remain relatively stable. Overall mortality rate in the G20 countries is projected to decline from 40.86 per 100,000 to 38.81 per 100,000. With respect to DALY rate, in China, the rate among females is projected to decrease from 630.25 per 100,000 to 595.48 per 100,000, while the rates among males and the overall population are projected to decline from 892.47 per 100,000 and 744.04 per 100,000, respectively, to 683.19 per 100,000 and 610.33 per 100,000. In G20 countries, the DALY rate among males is projected to decrease from 1042.92 per 100,000 to 941.16 per 100,000, whereas the rate among females is expected to remain relatively stable. Overall DALY rate in G20 countries is projected to decline from 871.04 per 100,000 to 825.24 per 100,000. Overall YLL rates are projected to decline in both China and G20 countries, although the female YLL rates in G20 countries is projected to remain stable. In contrast, overall YLD rates are projected to increase in both China and G20 countries, although sex-specific trends vary in China.

**Table 2:** Projections of incidence rates, mortality rates, and DALYs for COPD in China and G20 countries in 2024 and 2028

location	Measure	Gender	2024	2028
China	incidence	both	200.16 (199.25–201.07)	213.14 (199.85–226.43)
		female	186.79 (185.69–187.9)	191.67 (179.56–203.78)
		male	213.72 (212.92–214.52)	221.73 (208.09–235.38)
	prevalence	both	2398.58 (2389.95–2407.21)	2556.65 (2393.79–2719.52)
		female	2304.01 (2292.47–2315.56)	2248.98 (2143.75–2354.22)
		male	2455.24 (2445.51–2464.96)	2435.71 (2260.91–2610.52)
	mortality	both	43.97 (40.66–47.27)	35.84 (16.81–54.87)
		female	37.71 (34.54–40.89)	34.61 (11.09–58.14)
		male	53.87 (49.04–58.7)	43.67 (20.13–67.21)
	YLDs	both	105.72 (105.28–106.17)	107.8 (100.72–114.88)
		female	105.65 (105.1–106.2)	103.47 (97.95–108.99)
		male	105.69 (105.16–106.21)	107.34 (100.14–114.54)
	YLLs	both	638.41 (589.81–687)	505.44 (211.02–799.86)
		female	524.78 (479.88–569.68)	486.38 (153.4–819.35)
		male	786.83 (719.07–854.59)	577.45 (203.61–951.29)
	DALYs	both	744.04 (695.24–792.84)	610.33 (311.74–908.92)
		female	630.25 (585.22–675.28)	595.48 (261.53–929.43)
		male	892.47 (824.55–960.39)	683.19 (306.25–1060.14)
G20	incidence	both	199.03 (198.45–199.61)	204.43 (197.69–211.17)
		female	185.84 (185.25–186.43)	189.97 (184.57–195.37)
		male	214.69 (214.06–215.32)	217.81 (210.48–225.14)
	prevalence	both	2525.24 (2519.52–2530.96)	2561.81 (2491.31–2632.31)
		female	2446.06 (2439.81–2452.32)	2509.85 (2428.54–2591.16)
		male	2631.45 (2624.94–2637.97)	2650.49 (2567.61–2733.36)
	mortality	both	40.86 (38.86–42.86)	38.81 (31.44–46.18)
		female	33.15 (31.49–34.81)	33.15 (26.94–39.36)
		male	50.75 (48.15–53.36)	45.56 (36.27–54.85)
	YLDs	both	170.7 (170.23–171.16)	180.58 (172.26–188.89)
		female	161.2 (160.81–161.59)	169.72 (162.53–176.91)
		male	181.51 (180.9–182.13)	188.36 (178.39–198.33)
	YLLs	both	701.17 (666.03–736.31)	655.56 (529.78–781.33)
		female	564.6 (536.53–592.66)	564.6 (460.07–669.13)
		male	861.85 (815.91–907.79)	760.25 (603.25–917.25)
	DALYs	both	871.04 (835.42–906.66)	825.24 (697.3–953.18)
		female	725.00 (696.57–753.43)	725.00 (618.95–831.05)
		male	1042.92 (996.45–1089.38)	941.16 (781.61–1100.71)

### Two-sample MR analysis

**Figure 6:**
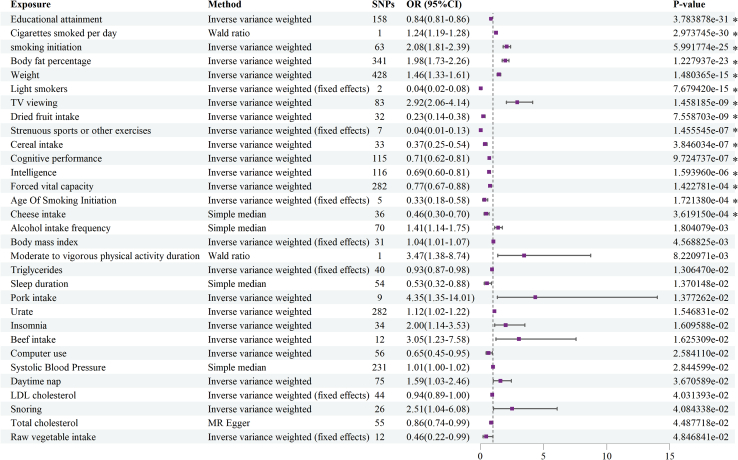
Analysis of the relationship between the variable factors and the risk of COPD Asterisks indicate associations that remained statistically significant after Bonferroni correction. OR, odds ratio; CIs, confidence intervals.

Among the modifiable exposures, a total of 31 factors were ultimately included to investigate their potential causal associations with COPD. The causal relationships between these factors and COPD risk are illustrated in Figure 6. After Bonferroni correction, 15 exposures remained significantly associated with the outcome. Educational attainment was associated with a lower risk (odds ratio [OR] = 0.84, 95% CI: 0.81–0.86), whereas smoking-related traits, including cigarettes smoked per day (OR = 1.24, 95% CI: 1.19–1.28) and smoking initiation (OR = 2.08, 95% CI: 1.81–2.39), were associated with increased risk. Adiposity-related traits, including body fat percentage (OR = 1.98, 95% CI: 1.73–2.26) and weight (OR = 1.46, 95% CI: 1.33–1.61), also showed robust positive associations. In addition, TV viewing was associated with increased risk (OR = 2.92, 95% CI: 2.06–4.14). In contrast, light smoking, dried fruit intake, strenuous sports or other exercises, cereal intake, cognitive performance, intelligence, forced vital capacity, later age of smoking initiation, and cheese intake were inversely associated with the outcome. Associations with alcohol intake frequency, body mass index, triglycerides, sleep duration, pork intake, urate, insomnia, beef intake, computer use, systolic blood pressure, daytime nap, LDL cholesterol, snoring, total cholesterol, and raw vegetable intake did not survive Bonferroni correction.

## Discussion

To our knowledge, this is a study that combines GBD 2023-based burden analysis with two-sample MR analysis to provide complementary evidence on COPD from both epidemiological and genetic perspectives. Specifically, the GBD 2023 analysis characterizes the temporal and geographic patterns of COPD burden in China and G20 countries, whereas the MR analysis explores potential causal associations between selected modifiable exposures and COPD risk. Although these two analytical components do not establish a direct mechanistic link between population-level burden trends and genetic causal estimates, their combination may help identify high-burden populations and exposures that warrant further investigation for COPD prevention. Our findings indicate that from 1990–2023, the ASMR, ASDR, and age-standardized YLL rates declined markedly in both China and the G20 countries, with consistently higher rates observed in males than in females. Notably, however, this downward trend showed a rebound after 2020, representing a concerning shift that warrants further attention. In contrast, the absolute numbers of incident and prevalent cases, as well as YLDs, continued to increase over time, which may be partly attributable to population aging.^10^^,^^11^ Overall, the temporal patterns of COPD burden in China and G20 countries are broadly consistent with previous reports.^12^^,^^13^ Taken together, the overall decline in ASR suggests that substantial progress has been made in COPD prevention and control across G20 countries, including China. These improvements may be largely attributable to the implementation of tobacco control and environmental protection policies. Smoking is one of the most important risk factors for COPD, and the WHO has emphasized that reducing exposure to tobacco smoke is a key strategy for preventing and mitigating COPD burden.^14^ In response, G20 countries have adopted a range of tobacco control measures in accordance with the WHO Framework Convention on Tobacco Control (FCTC), including increased tobacco taxation, smoke-free policies, health warning labels, advertising restrictions, and smoking cessation services.^3^^,^^14^ In terms of environmental protection, several G20 economies have incorporated air quality improvement into their national development strategies in recent years.^15^^,^^16^ For example, China’s Clean Air Policy on Criteria Air Pollutants and Health Risks has substantially reduced population-level exposure to air pollution.^17^ The implementation and scaling up of these policies have improved environmental quality in many countries and regions, thereby contributing to a reduction in COPD-related risk factors.

The disaggregated comparison with Germany, Japan, India, and Brazil provides additional policy-relevant insights beyond the overall G20 average. China’s relatively faster decline in COPD burden compared with India and Brazil may partly reflect the combined effects of strengthened chronic disease management, improved primary healthcare capacity, tobacco control efforts, air pollution control, and broader socioeconomic development. In recent years, China has increasingly emphasized COPD prevention and management within the Healthy China framework, including improved screening and management capacity in primary healthcare settings. For example, national efforts have promoted early detection among high-risk populations, expanded the use of spirometry in primary care, and strengthened training for healthcare workers, which may have contributed to earlier diagnosis and more standardized disease management.^18^ By contrast, India and Brazil face challenges typical of emerging economies, including large populations, regional inequalities, environmental exposures, and unequal access to early diagnosis and long-term respiratory care. India has implemented national programs targeting noncommunicable diseases, air pollution, and tobacco control, yet persistent air pollution, tobacco exposure, and uneven healthcare access may hinder reductions in COPD burden.^19^ Brazil has achieved substantial progress in tobacco control and has incorporated CRDs into its national noncommunicable disease strategy. However, socioeconomic inequality, lack of specialist care, and limited integration of COPD management into primary care may continue to constrain further progress.^20^ The remaining differences between China and high-income countries such as Germany and Japan may provide further guidance for policy improvement. Germany has implemented structured disease management programs for chronic diseases, including COPD, which emphasize guideline-based care, patient education, coordinated follow-up, and quality assurance.^21^ Such integrated models may help improve long-term disease management and reduce adverse outcomes. Japan has incorporated COPD into Health Japan 21, with goals related to COPD awareness and smoking reduction, and has issued clinical guidance for COPD management.^22^ Taken together, these country-specific comparisons suggest that China should continue reducing major risk exposures, especially tobacco use and air pollution, while further strengthening standardized long-term management, pulmonary rehabilitation, primary care follow-up, and equitable access to respiratory healthcare. Since the emergence of COVID-19 at the end of 2019, global healthcare systems and clinical practices have undergone profound changes.^23^ Previous studies have reported that the COVID-19 pandemic substantially affected the prevention and management of CRDs, including COPD.^24^ In our analysis, a slight rebound in ASDR, ASMR, and age-standardized YLL rates was observed in G20 countries after 2020, which may partly reflect the impact of the COVID-19 pandemic on the burden of COPD.^24^ Patients with COPD have limited baseline pulmonary reserve, and increased expression of angiotensin-converting enzyme 2 (ACE2) receptors in the small airways may facilitate SARS-CoV-2 entry. Following infection, an exaggerated immune response and cytokine storm can occur, increasing the risk of severe pneumonia and leading to poorer clinical outcomes.^25^^,^^26^ Moreover, COPD management faced considerable challenges during the pandemic. Face-to-face consultations were reduced, pulmonary rehabilitation programs and home-visit services were restricted, and patients who would typically seek medical care during disease exacerbations may have delayed hospital visits due to fear of viral exposure, resulting in postponed treatment and potentially worse outcomes.^25^ Although the COVID-19 pandemic may have contributed to increased COPD burden through healthcare disruptions, delayed diagnosis and treatment, reduced pulmonary rehabilitation, and exacerbation of underlying respiratory diseases, our analysis cannot establish a direct causal effect of the pandemic. In the sensitivity analysis excluding 2020–2023 data, Joinpoint regression and ARIMA models based on the 1990–2019 pre-pandemic period suggested that COPD burden indicators would generally continue to decline during 2020–2023. However, the observed GBD 2023 estimates showed a slight rebound in several indicators, suggesting a deviation from the pre-pandemic trend. Nevertheless, this discrepancy may reflect not only true epidemiological changes but also potential data or methodological artifacts, including changes in death certification practices, diagnostic shifts, underascertainment of COPD during lockdowns, changes in healthcare-seeking behavior, disruptions in routine COPD management, cause-of-death redistribution, and updates in the GBD 2023 estimation framework. Future studies using formal interrupted time-series designs, independent national surveillance data, and longer post-pandemic follow-ups are needed to clarify whether the post-2020 rebound represents a sustained epidemiological change or a data-related artifact.

Sex disparities in COPD burden have long been a focus of epidemiological research. Previous studies have consistently shown that the burden of COPD is higher in males than in females.^27^ Although our findings confirm this pattern, we observed that the sex gap has gradually narrowed over time. Historically, the proportion of current and former smokers has been substantially higher among men than women, and occupational exposures have been more concentrated in male-dominated labor sectors in many countries, contributing to the higher COPD burden among men.^28^^,^^29^^,^^30^ However, the narrowing sex disparity in recent years may be attributable to several factors. First, the implementation of tobacco control policies has contributed to a reduction in smoking prevalence among men.^3^^,^^14^ Second, with the acceleration of industrialization and urbanization, women’s participation in the workforce—and consequently their exposure to occupational risk factors—has increased.^27^ In addition, improvements in healthcare accessibility and heightened health awareness may have influenced the observed changes in sex differences. The expansion of early screening programs and standardized treatment for COPD has likely improved the identification and diagnosis of female patients, thereby revealing a previously underestimated disease burden among women.^31^^,^^32^ Meanwhile, advances in medical research have increasingly emphasized sex-specific differences in disease presentation, progression, and treatment response, providing a foundation for more precise and individualized intervention strategies.

We further applied MR analysis to identify modifiable factors that may reduce the burden of COPD at the individual level. MR is an epidemiological approach that uses genetic variants randomly allocated during meiosis as instrumental variables (IVs) to infer causal relationships between exposures and outcomes. By leveraging the random assortment of alleles, this method minimizes reverse causation and confounding from sociodemographic or behavioral factors commonly encountered in observational studies, thereby reducing bias due to residual confounding.^33^ Smoking initiation was strongly associated with an increased risk of COPD, and a higher number of cigarettes smoked per day likewise indicated elevated risk, consistent with the Global Initiative for Chronic Obstructive Lung Disease (GOLD) guidelines.^34^ Notably, a later age at smoking initiation showed a significant protective association, suggesting that delaying or preventing smoking initiation may substantially reduce COPD risk. These findings underscore that, beyond reducing smoking intensity, postponing or avoiding smoking initiation is of critical importance. Adiposity-related indicators, including body fat percentage and body weight, demonstrated positive causal associations with COPD risk. Obesity may adversely affect COPD outcomes through reduced chest wall compliance and restricted lung volumes, chronic low-grade inflammation, and metabolic dysregulation.^35^ Educational attainment emerged as one of the most robust protective factors, and cognitive performance and intelligence showed consistent inverse associations with COPD risk. These findings align with recent MR studies suggesting a potential causal protective effect of higher educational attainment on COPD incidence and hospitalization risk, possibly mediated in part through pathways such as reduced smoking and improved weight control.^36^ Although the MR analysis provided genetic evidence supporting potential causal associations between several exposure factors and COPD, these findings should be interpreted with caution. Several associations remained statistically significant after sensitivity analyses and multiple-testing correction; however, some estimates showed relatively large effect sizes, such as the association between television watching and COPD risk. Given that COPD is a complex multifactorial disease influenced by smoking, environmental exposures, aging, genetic susceptibility, socioeconomic status, and healthcare access, such large estimates for behavioral exposures may not necessarily indicate a direct causal relationship. Instead, they could reflect horizontal pleiotropy, residual confounding, or limited specificity of the genetic instruments. In addition, the ancestry background of the genome-wide association study (GWAS) data should be considered when interpreting the MR results. The GWAS summary statistics used in this study were obtained from the FinnGen consortium, which predominantly includes individuals of European, particularly Finnish, ancestry. Therefore, although the epidemiological analysis evaluated the COPD burden in China and G20 countries, the MR findings may not be fully generalizable to Chinese or other non-European populations. Population differences in genetic architecture, allele frequencies, linkage disequilibrium patterns, environmental exposures, lifestyle behaviors, and healthcare systems may influence the applicability of these causal estimates. Accordingly, caution is warranted when extrapolating these MR results to non-European populations or when using them to inform COPD-prevention strategies in China and other G20 countries. Future studies using GWAS data from East Asian and other diverse populations, together with independent validation and mechanistic investigations, are needed to confirm these findings and improve their generalizability.

In summary, this study provides a comprehensive analysis and projection of the burden and trends of COPD in China and G20 countries. Over the past 32 years, ASRs have declined in both China and the G20, likely reflecting the implementation of preventive strategies, advances in healthcare, and improvements in socioeconomic conditions. However, the observed rebound in ASDR and ASMR after 2020 warrants further investigation to clarify its underlying causes and long-term implications. Moreover, our MR findings suggest potential causal associations between several modifiable factors and COPD risk. Given the potential influence of horizontal pleiotropy, weak instrument bias, and uncertainty related to large effect estimates, these MR findings should be interpreted cautiously and require replication in independent GWAS datasets and diverse populations. Therefore, targeted interventions aimed at reducing exposure to these controllable risk factors may be considered in future prevention strategies. The results of this study may provide valuable evidence to inform public health interventions and clinical strategies aimed at reducing the burden of COPD in China and other G20 countries.

### Limitations of the study

Despite its strengths, this study has several limitations that should be acknowledged. First, although the data were obtained from the GBD database and underwent rigorous cleaning and standardization procedures, some degree of uncertainty remains with respect to data quality control, including variations in data collection methods, sources, processing, coding practices, and healthcare accessibility across regions. Consequently, discrepancies between GBD estimates and real-world data may persist. Second, this study did not comprehensively account for other potential risk factors associated with COPD, such as detailed genetic background, specific occupational exposures, and environmental influences. Another limitation is related to the ARIMA forecasting model. Although ARIMA models are commonly used for time-series forecasting, they primarily rely on historical temporal patterns and do not explicitly incorporate population aging, cohort effects, changes in smoking prevalence, environmental exposure patterns, healthcare improvements, or future prevention and treatment strategies. Therefore, ARIMA-based projections may become increasingly uncertain as the forecasting horizon extends, particularly for chronic diseases such as COPD with long latency and progressive disease courses. Long-term extrapolation may lead to error accumulation, unstable prediction intervals, or biologically implausible estimates. Fourth, although GBD 2023 offers standardized estimates for cross-country comparisons, variations in data availability, diagnostic capacity, death certification, and healthcare systems across G20 countries may affect comparability. In data-sparse settings, estimates rely more heavily on modeling and cause-of-death redistribution, introducing additional uncertainty. Therefore, comparisons between China and the overall G20 average should be interpreted cautiously, as the average may mask substantial heterogeneity among countries. These factors warrant further investigation in future studies to provide a more complete understanding of COPD etiology and burden.

## Resource availability

### Lead contact

Further information and requests for resources should be directed to and will be fulfilled by the lead contact, Chen Wenyu (00135116@zjxu.edu.cn).

### Materials availability

This study did not generate new unique reagents.

### Data and code availability

• Data are available in a public, open access repository. The GBD data are publicly accessible via the Global Health Data Exchange (GHDx) tool, and the MR summary statistics are from FinnGen, which is accessible.
• All original code has been deposited at GitHub and is publicly available as of the date of publication. The repository link is listed in the key resources table.
• Any additional information required to reanalyze the data reported in this paper is available from the lead contact upon request.

## Acknowledgments

This work was supported by Medical and Health Science Program of Zhejiang Province (no. 2025HY1192), Key Construction Disciplines of Provincial and Municipal Co-construction of Zhejiang (no. 2023SSGJ002), Morning Star Talent Development Program (no. 2025QMX019), Traditional Chinese Medicine Clinical Research Program Project (no. 2026ZL0894), National Oncology Clinical Key Speciality (no. 2023GJZK001), and Jiaxing Key Laboratory of Precision Treatment for Lung Cancer (no. 2023GJZK001). We acknowledge the 10.13039/100014544Institute for Health Metrics and Evaluation (IHME) and the Global Burden of Disease collaborators for providing access to the GBD 2023 data.

## Author contributions

Conception and design, H.S. and C.W.; provision of study materials, C.W., Z.M., and T.X.; collection and assembly of data, H.S. and T.X.; data analysis and interpretation, H.S.; manuscript writing, H.S. and C.W.; final approval of manuscript, all authors.

## Declaration of interests

The authors declare no competing interests.

## Declaration of generative AI and AI-assisted technologies in the writing process

During the preparation of this work, we used DeepSeek for language translation and linguistic polishing. After using this tool, we reviewed and edited the content as needed and take full responsibility for the content of the publication. We confirm that no generative AI or AI-assisted technologies were used for data analysis, statistical modeling, result interpretation, or the generation of scientific conclusions.

## STAR★Methods

### Key resources table

**Table:** 

REAGENT or RESOURCE	SOURCE	IDENTIFIER
**Deposited data**		

Global Burden of Disease Study 2023	Data from the GBD study in 2023 can be accessed using the Global Health Data Exchange (GHDx) query tool	https://vizhub.healthdata.org/gbd-results/
Code used for this study	This paper	https://github.com/Chenwenyu1/Original-code2

**Software and algorithms**		

R version 4.4.2	R Core Team	https://www.r-project.org/
Joinpoint Regression Program (Version 5.2.0)	National Cancer Institute	https://surveillance.cancer.gov/joinpoint/

### Experimental model and study participant details

This study is a secondary analysis using publicly available, geographically aggregated data from the Global Burden of Disease (GBD) 2023 database and summary-level genome-wide association study (GWAS) data for two-sample Mendelian randomization analyses. The GBD component assessed the burden of chronic obstructive pulmonary disease (COPD) in China and G20 countries from 1990 to 2023, whereas the Mendelian randomization component evaluated genetically proxied associations between selected risk factors and COPD. This study did not involve the direct enrollment of human participants, nor did it use any animal, cellular, or other experimental models. All GBD data analyzed in this study were anonymized and aggregated at the national or regional level. The GWAS data used for Mendelian randomization were obtained from publicly available summary statistics, without access to individual-level genetic or phenotypic information. Because this study used only publicly available, aggregated, anonymized, or summary-level data, individual patient consent was not required. Ethical approval and informed consent for the original GBD and GWAS source studies were obtained by the original investigators according to their respective institutional requirements. No additional institutional ethical approval was required for the present secondary analysis under local regulations. Patients and the public were not involved in the design, conduct, analysis, or reporting of this study. Where applicable, this study follows the Guidelines for Accurate and Transparent Health Estimates Reporting (GATHER) framework for secondary analyses of global health estimates. In accordance with the journal’s guidelines on sex and gender reporting, GBD analyses were stratified by biological sex, and sex-specific findings are reported throughout the Results, including analyses of age-, sex-, and region-specific COPD burden. The Mendelian randomization analyses were based on the sex information available in the original GWAS summary datasets, and no additional participant-level sex or gender data were collected in this study.

### Method details

The GBD 2023 study, led by the Institute for Health Metrics and Evaluation (IHME), is a comprehensive initiative that analyzes the incidence, prevalence, mortality, years lived with disability (YLDs), years of life lost (YLLs), disability-adjusted life years (DALYs), health-adjusted life expectancy (HALE), and 88 modifiable risk factors across 375 diseases and injuries.^8^^,^^9^ It provides a comprehensive assessment of health losses from 1990 to 2023. This study utilizes the GBD tool to retrieve and analyze data on COPD incidence, prevalence, mortality, YLDs, YLLs, DALYs for China and G20 countries from 1990 to 2023. The COPD diagnostic codes used in this study include ICD-10: J41-J42.4, J43-J44.9, ICD-9: 491–492.9, 496–499. Data for this analysis were collected using the Global Health Data Exchange (GHDx) query tool (http://ghdx.healthdata.org/). Since the data used in this study is publicly available, no additional ethical approval was required for this research.

The summary data for the genome-wide association study (GWAS) of COPD used in this study was sourced from the FinnGen consortium.^37^ The variables included in this analysis were classified into the following categories: (i) Metabolic factors, including obesity indicators, fasting blood glucose, fasting insulin, glycated hemoglobin, blood lipids, and uric acid levels; (ii) Physical conditions, such as height, weight, blood pressure, and lung function (forced vital capacity); (iii) Lifestyle factors, including smoking, alcohol consumption, and dietary intake; (iv) Sleep conditions, including insomnia, daytime napping, and snoring; (v) Physical activity, including sedentary behavior, moderate-to-vigorous physical activity (MVPA), and vigorous physical activity (VPA). The FinnGen study protocol was approved by the Helsinki and Uusimaa Hospital District Ethical Committee (approval number HUS/990/2017), and participants provided informed consent.^37^

### Quantification and statistical analysis

We extracted data from the GBD 2023 study on COPD incidence, prevalence, mortality, YLDs, YLLs, and DALYs for China and G20 countries, along with the corresponding age-standardized incidence rate (ASIR), age-standardized prevalence rate (ASPR), age-standardized mortality rate (ASMR), age-standardized DALY rate (ASDR), age-standardized YLDs rate and age-standardized YLLs rate. All estimates are presented with 95% uncertainty intervals (UIs). Joinpoint regression models were applied to calculate the annual percent change (APC), together with their corresponding 95% confidence intervals (CIs), in order to identify significant long-term changes in COPD trends in China and the G20 countries. Segmented trend analysis was performed using Joinpoint regression models. The maximum number of joinpoints was set to 4. The optimal number of joinpoints and the final segmented regression model were selected using the built-in permutation test in the Joinpoint software, with the Bayesian information criterion (BIC) also considered to balance model fit and model parsimony. To account for potential heteroscedasticity in the time-series data, heteroscedasticity-robust estimation was applied, thereby relaxing the assumption of constant variance across different time periods and improving the reliability of the fitted temporal trends in COPD burden indicators.^38^^,^^39^ The direction of the trend was determined based on the APC derived from the final model. Autoregressive integrated moving average (ARIMA) models were used to project COPD incidence, prevalence, mortality, YLDs, YLLs, and DALYs in China and the G20 countries from 2024 to 2028.^40^ The future burden of COPD was projected using autoregressive integrated moving average (ARIMA) models. Model selection was performed using the forecastauto.arima function in R. To improve the robustness of model selection, both stepwise search and approximation were disabled, allowing an exhaustive search across candidate ARIMA models. The stationarity of each time series was assessed using the unit root tests implemented in the auto.arima procedure, and the optimal differencing order (d) was automatically determined. The autoregressive order (p) and moving average order (q) were selected according to the corrected Akaike information criterion (AIC). Because the GBD estimates were annual data, no seasonal component was included in the ARIMA models. Model adequacy was evaluated through residual diagnostic tests. The Ljung–Box test was used to assess whether the residuals approximated white noise, and the Shapiro–Wilk test was used to examine residual normality. In addition, model fitting performance was assessed using the root-mean-square error (RMSE), mean absolute error (MAE), AIC, and BIC. Bootstrap resampling was performed 1,000 times to project COPD burden indicators over the subsequent 5 years, from 2024 to 2028, and the corresponding 95% prediction intervals were calculated. The specific modeling procedures were consistent with those reported in previous studies.^41^ Statistical analyses and data visualization were performed using R software (version 4.4.2) and the Joinpoint Regression Program (version 5.2.0). A two-sided *p* value <0.05 was considered statistically significant.

To assess whether the observed post-2020 rebound was robust or potentially influenced by pandemic-related data artifacts, we conducted a sensitivity analysis excluding data from 2020 to 2023. Joinpoint regression was repeated using only pre-pandemic data from 1990 to 2019. In addition, ARIMA models were re-fitted using the 1990–2019 time series to generate counterfactual projections for COPD burden indicators during 2020–2023. These projected estimates were then compared with the observed GBD 2023 estimates for the same period.

We performed a two-sample MR analysis to evaluate the potential causal associations between genetically predicted exposures and COPD outcomes. For each exposure, single-nucleotide polymorphisms (SNPs) significantly associated with the exposure were selected as instrumental variables (IVs). After harmonizing effect alleles between exposure and outcome datasets, ratio estimates were calculated for each SNP. The proportion of variance in the exposure explained by each SNP was estimated using the R^2^ statistic,^42^ and instrument strength was assessed using the F statistic.^43^ As the primary analytical approach, we applied the inverse-variance weighted (IVW) method^44^ to combine effect estimates across multiple SNPs and obtain an overall causal estimate. A fixed-effects IVW model was used when Cochran’s Q test indicated no substantial heterogeneity. For exposures instrumented by a single SNP, where multi-SNP meta-analysis was not feasible, the Wald ratio method was used to estimate the causal effect. To assess the robustness of the findings and minimize potential bias arising from invalid instruments or horizontal pleiotropy, we conducted several complementary MR sensitivity analyses, including the simple median method^45^ and MR-Egger regression.^46^ All causal estimates were reported as odds ratios (ORs) with corresponding 95% CIs on a consistent effect scale.^33^ To assess instrument strength, F statistics were calculated for each instrumental variable, and SNPs with F statistics <10 were excluded to reduce potential weak instrument bias. To account for multiple comparisons across 31 exposure factors, Bonferroni correction was applied, and a *p* value < 0.05/31 was considered statistically significant after correction. Associations with *p* values between 0.05/31 and 0.05 were regarded as suggestive evidence. Heterogeneity among instrumental variables was assessed using Cochran’s Q test, and potential directional horizontal pleiotropy was evaluated using the MR-Egger intercept test (Tables S1 and S2).

Artificial intelligence (DeepSeek) was used to assist with language translation and linguistic polishing. All scientific content, data analyses, and interpretations were performed independently by the authors.

## Supplemental information

Document S1. Figures S1–S4

Table S1. Instrumental variable characteristics and F statistics for the Mendelian randomization analysis

Table S2. Sensitivity analyses for Mendelian randomization: Heterogeneity and horizontal pleiotropy tests
