# Supplementary material for: COPD burden trends in China and G20 countries from GBD 2023 and Mendelian randomization
Source: iScience. 2026 Aug 10;29(8):117058. doi: 10.1016/j.isci.2026.117058 (PMC13486890; doi:10.1016/j.isci.2026.117058)
Supplement: Supplementary file 1 [file mmc1.pdf]

iScience, Volume *29*

Supplemental information

COPD burden trends in China and G20 countries from GBD 2023 and Mendelian randomization

Hu Shiyu, Tan Xiaoli, Zhao Miaomiao, and Chen Wenyu

Supplementary materials

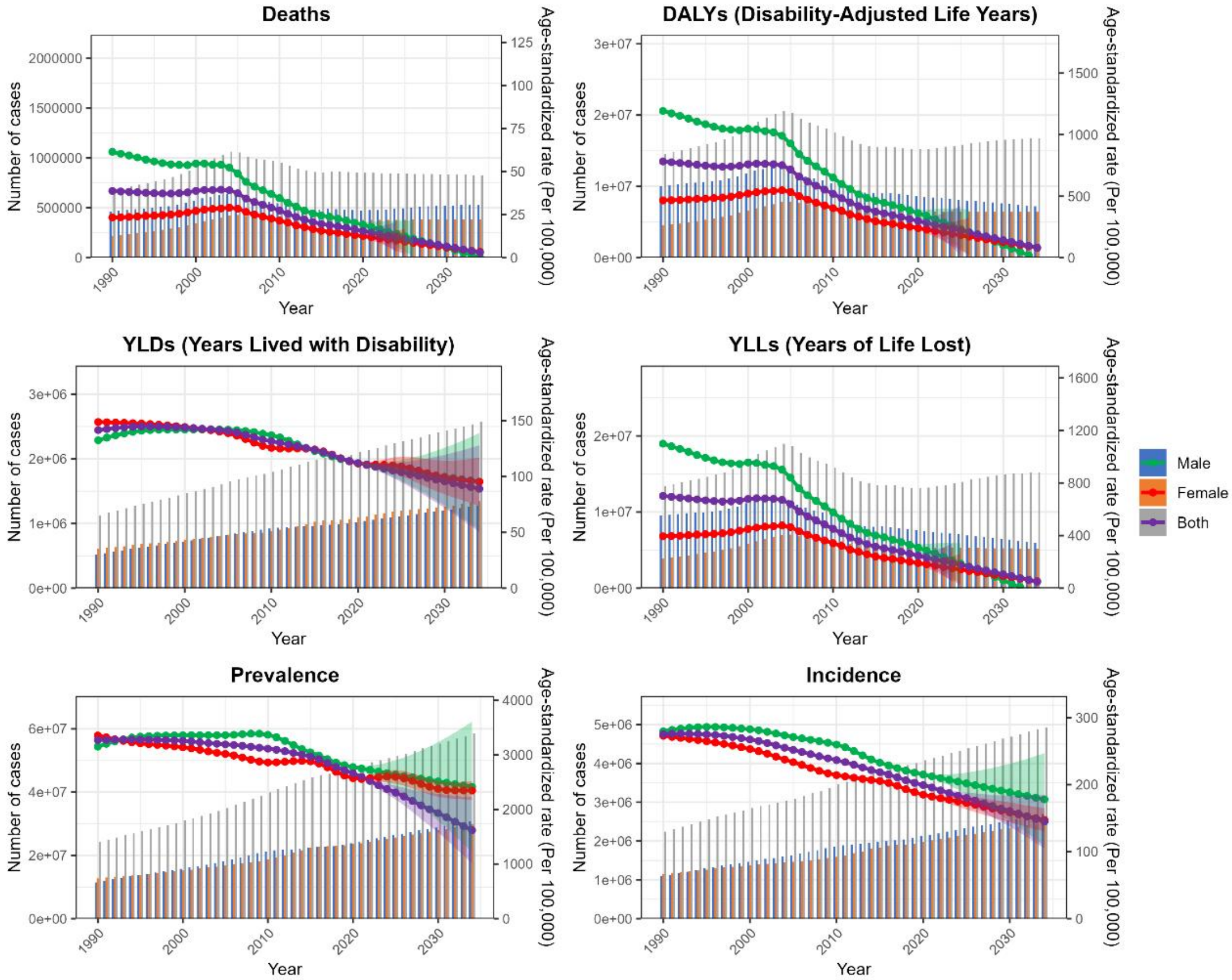

Figure S1 ARIMA projections based on pre-pandemic data from 1990 to 2019 in China. Bars indicate the absolute number of cases and correspond to the left y-axis, whereas lines indicate age-standardized rates per 100,000 population and correspond to the right y-axis. Error bars represent uncertainty intervals for the number of cases. Green indicates males, red indicates females, and purple indicates both sexes.

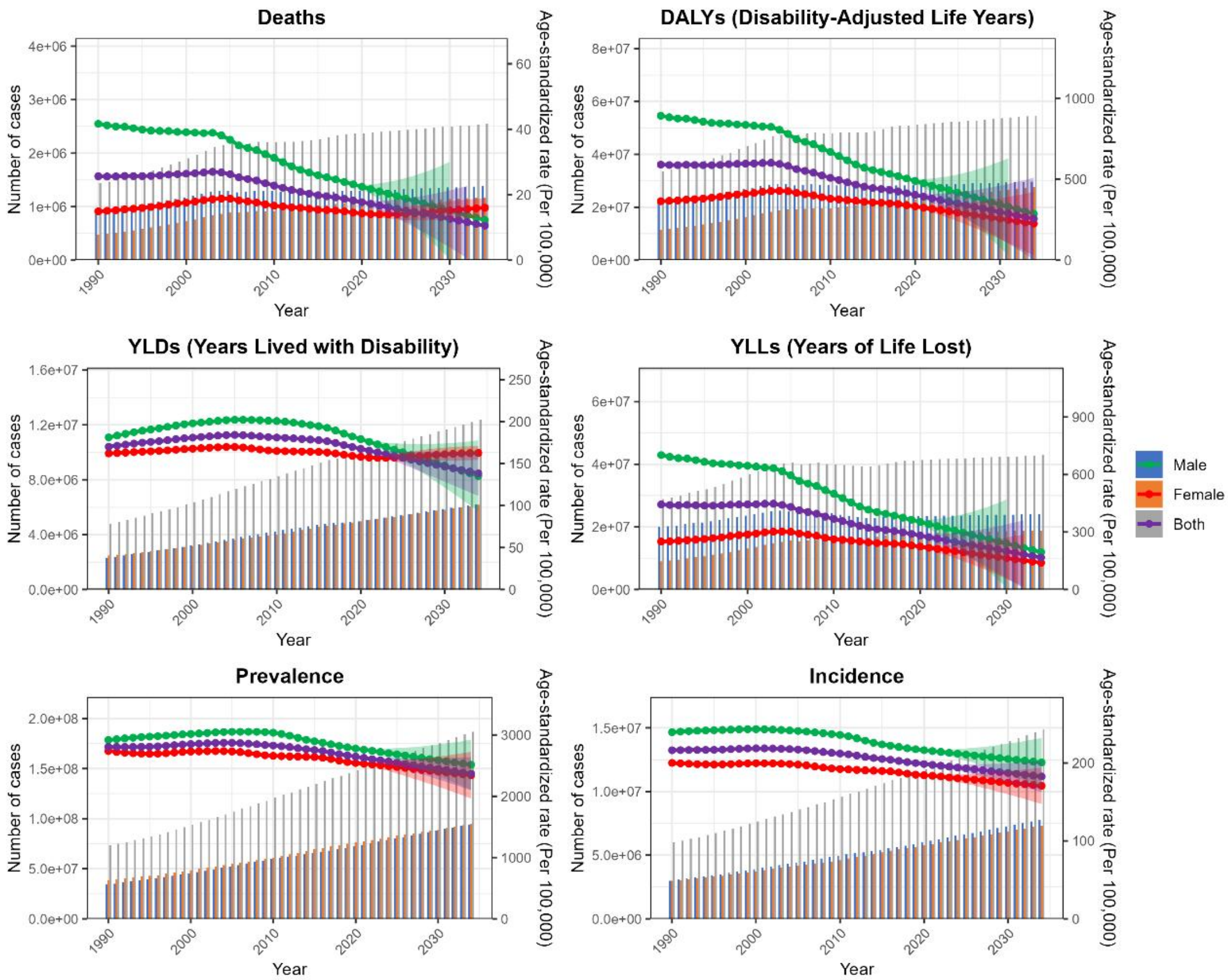

Figure S2 ARIMA projections based on pre-pandemic data from 1990 to 2019 in G20 Countries. Bars indicate the absolute number of cases and correspond to the left y-axis, whereas lines indicate age-standardized rates per 100,000 population and correspond to the right y-axis. Error bars represent uncertainty intervals for the number of cases. Green indicates males, red indicates females, and purple indicates both sexes.

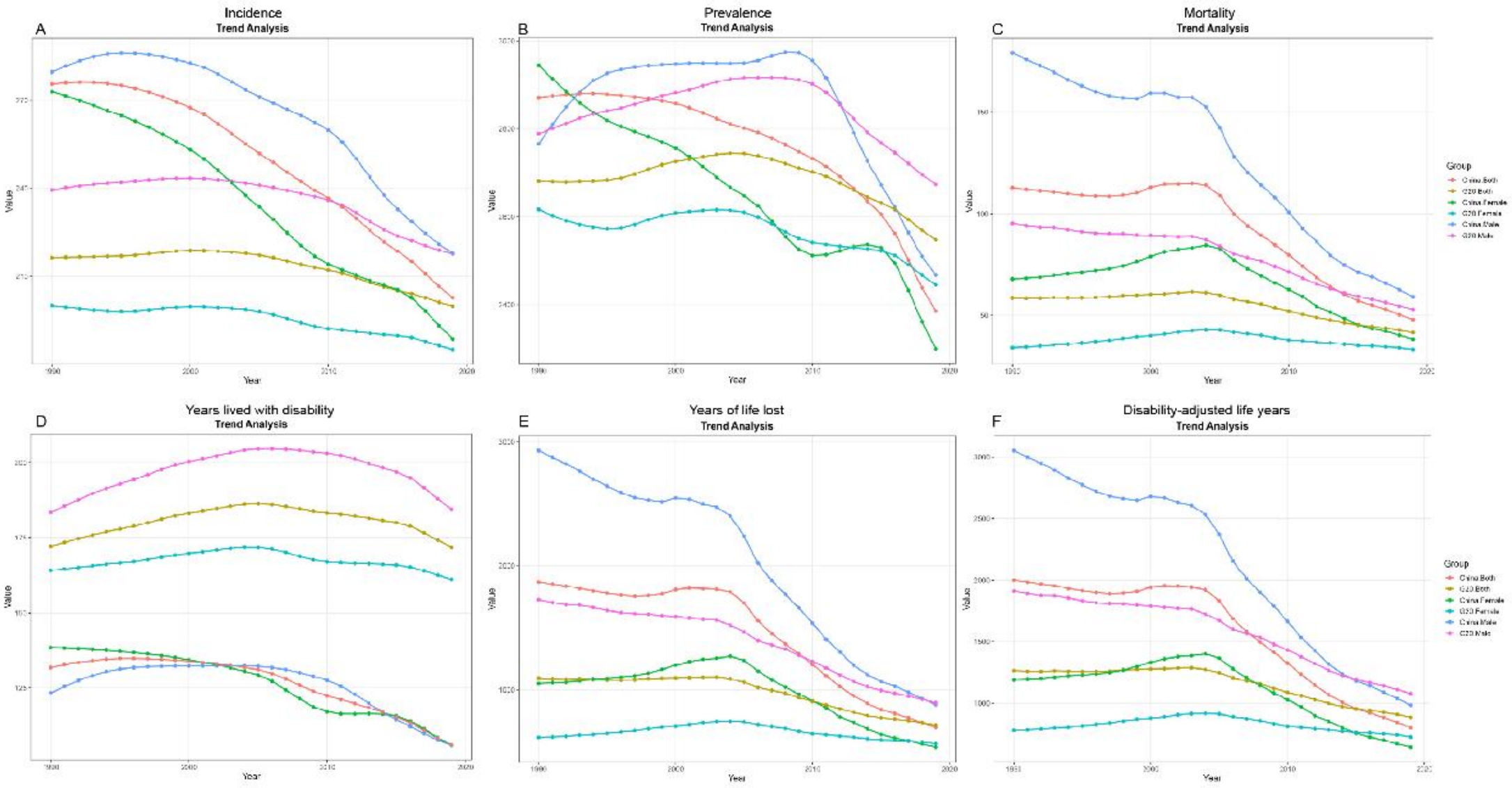

Figure S3 Joinpoint regression analysis based on pre-pandemic data from 1990 to 2019. Red, China both sexes; yellow, G20 both sexes; green, China females; cyan, G20 females; blue, China males; purple, G20 males.

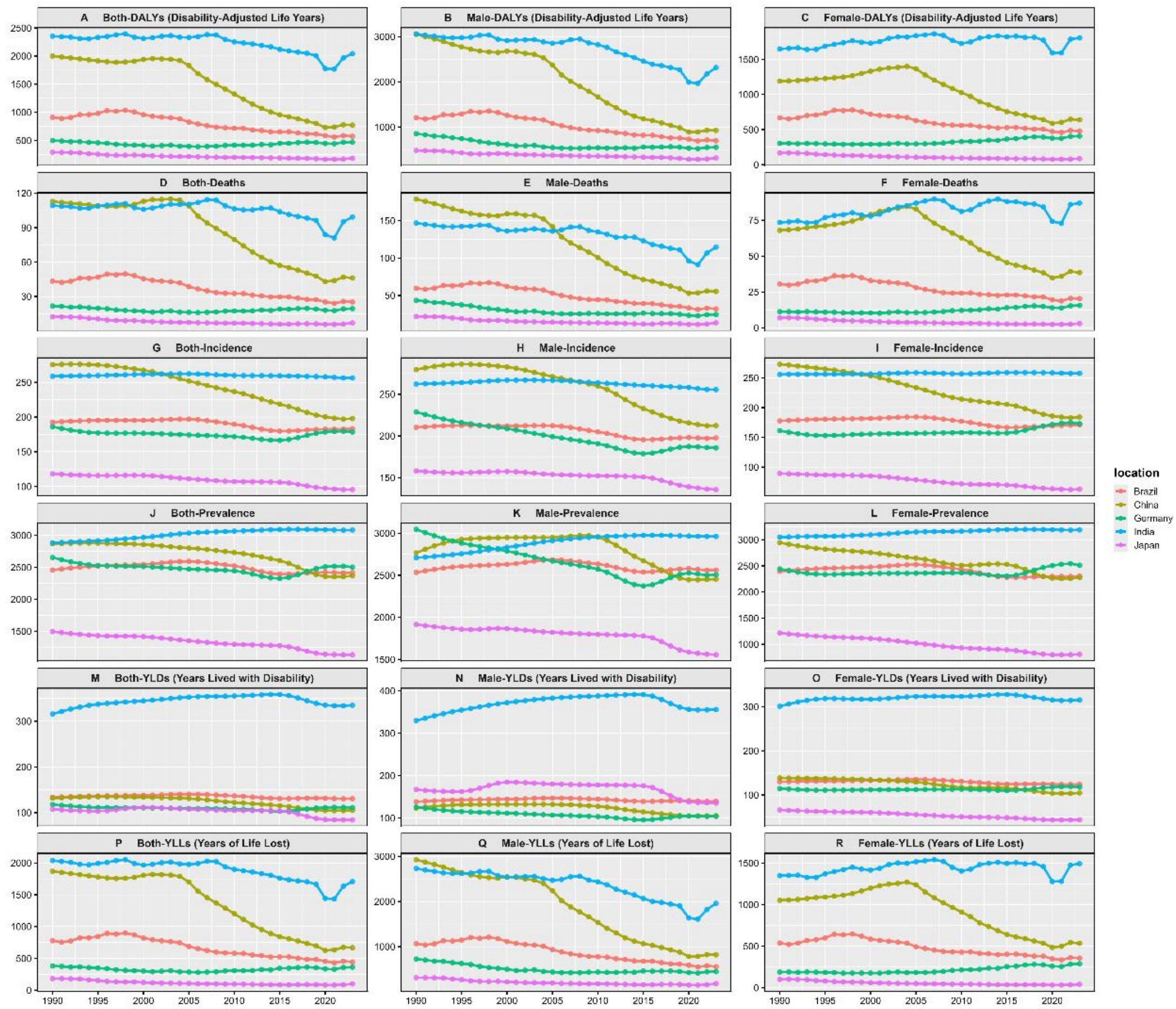

Figure S4 Comparison of COPD burden between China and selected G20 countries. Red, Brazil; yellow, China; green, Germany; blue, India; purple, Japan.
